# Naphthalene Tetrazole-Based Nickel Metal–Organic Framework as a Filler of Polycarbonate Membranes to Improve CO_2_ and H_2_ Separation

**DOI:** 10.1021/acsapm.4c00277

**Published:** 2024-04-01

**Authors:** Antonio Valverde-Gonzalez, Nastasiya Yuriychuk, M. Carmen Borrallo-Aniceto, Felipe Gándara, Marta Iglesias, Mar López-González, Eva M. Maya

**Affiliations:** † Departamento de Fronteras en Química de Materiales, Instituto de Ciencia de Materiales de Madrid (ICMM), CSIC, Sor Juana Inés de la Cruz, 3, Cantoblanco, Madrid 28049, Spain; ‡ Departamento de Química-Física de Polímeros, Instituto de Ciencia y Tecnología de Polímeros (ICTP-CSIC), Consejo Superior de Investigaciones Científicas, C/Juan de la Cierva 3, Madrid 28006, Spain

**Keywords:** mixed matrix membranes, nickel metal–organic framework, tetrazole-naphthalene linker, CO_2_/CH_4_ separation, H_2_/CH_4_ separation

## Abstract

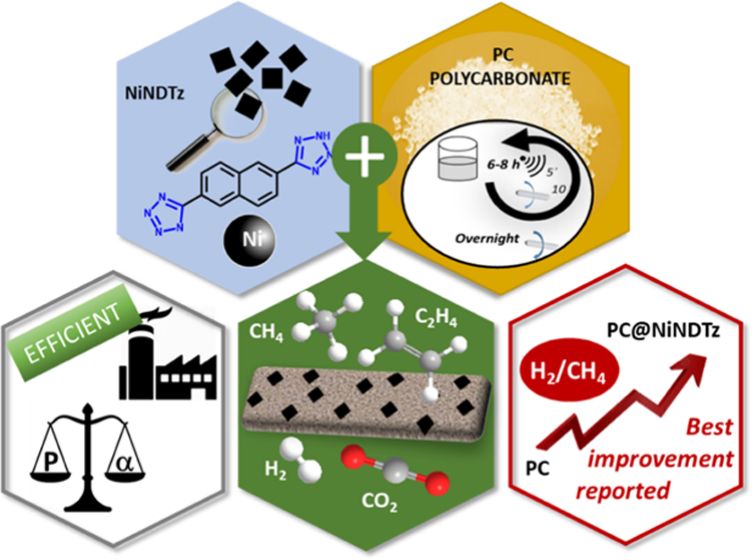

A tetrazole-naphthalene linker was used to prepare a nickel MOF (metal–organic framework) (**NiNDTz**) with interesting properties: a specific surface area *S*_BET_ of 320 m^2^g^–1^ (*S*_Langmuir_ 436 m^2^g^–1^), high thermal stability (Td_onset_ = 300 °C), and CO_2_ uptake of 1.85 mmolg^–1^, attributed to the tetrazole groups to be used as fillers in gas separation membranes. The role of these groups was crucial in the mechanical properties of mixed membranes prepared using polycarbonate as a polymer matrix, providing a very homogeneous filler distribution and also in the gas separation properties since a simultaneous increase in permeability and selectivity was achieved, especially in the hybrid membrane containing 20% filler (**PC@NiNDTz-20%**). This membrane exhibited an excellent balance between permeability (*P*) and selectivity (α) with an increase in the permeability of CO_2_ and H_2_, 177 and 185%, respectively, and improvements in the selectivity of these gases against greenhouse gases such as methane and ethylene (between 15 and 28% improvement). These results make this membrane competitive to deal with separations in which these gases are involved, and are of special interest for the H_2_/CH_4_ separation since it clearly improves the performance of pure PC and no better PC-based membranes have been reported in the literature for this separation.

## Introduction

**Figure 1:**
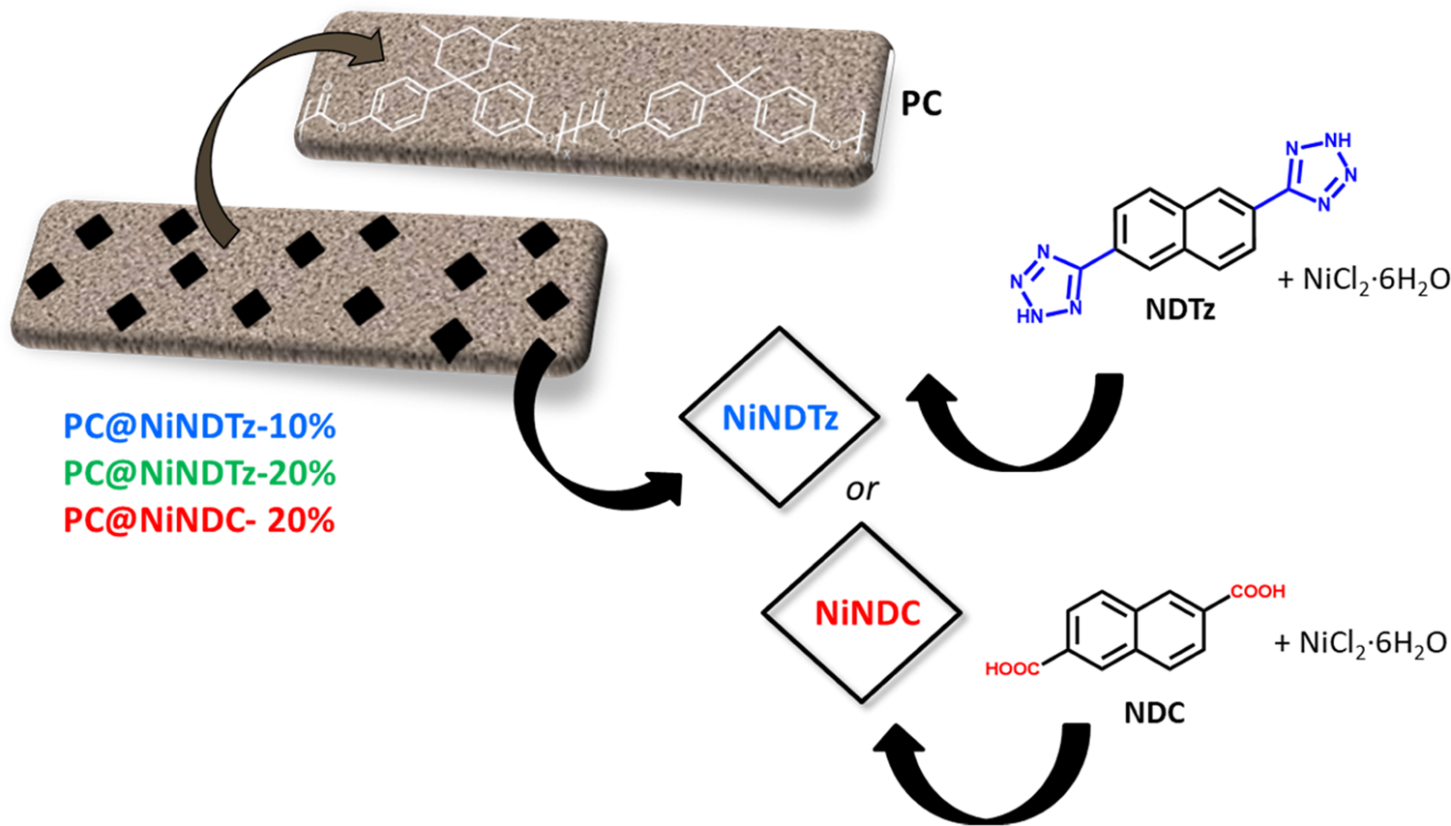
Representation of pristine polycarbonate (PC) and mixed-matrix membranes of PC and naphthalene-based Ni-MOFs from tetrazole (NDTz) or dicarboxylic acid (NDC) linkers (PC@NiNDTz and PC@NiNDC).

Metal–organic frameworks (MOFs) have been widely employed as fillers in mixed-matrix membranes (MMMs) for gas transport applications, owing to their excellent ability to separate gases due to their peculiar structures containing narrow and fixed pore sizes.^1,2^ Organic dicarboxylate linkers have typically been the most widely used to prepare MOFs.^1−4^ However, in the past decade, there has been an increasing interest in using tetrazoles as linkers to obtain MOFs because they have three special features: (1) the ability to form different structures due to their multifunctional coordination characteristics; (2) nonlinearity, which reduced the probability of interpenetration; and (3) N-rich character, which allows building frameworks with N-donor decorated channels.^5^ As a consequence, these structures result in strong metal–nitrogen bonds that endow high chemical and thermal stabilities to the frameworks.^6,7^ One of the typical applications for these tetrazole-based MOFs is to use them as sensors for both chemicals and metal ions,^8−17^ or recently as heterogeneous catalysts.^18−20^ However, as far as we know, the incorporation of tetrazole-based MOFs as fillers in mixed-matrix membranes has not yet been explored. Thus, in this work, we have reported for the first time the incorporation of 10 and 20% of naphthalene tetrazole-based Ni-MOFs (**NiNDTz**) into a commercial polycarbonate (PC) to obtain the corresponding mixed matrix membranes, **PC@NiNDTz-10%** and **PC@NiNDTz-20%** (Figure 1). This matrix, polycarbonate, was selected because besides its gas transport properties,^21^ it is a cheaper material compared to others such as polysulfone (PS), polyimides (PIs), or polymers of intrinsic porosity (PIMs). The gas transport properties of O_2_, N_2_, CO_2_, H_2_, and CH_4_ were studied to predict different gas separations of interest. Moreover, ethylene was also measured due to its importance in petrochemical and agricultural industries. In this sense, membranes with high CO_2_/C_2_H_4_ and H_2_/C_2_H_4_ separation selectivity need to be developed to achieve competitive processes at the economic and industrial levels.

To establish the effect of tetrazole groups on membrane properties and gas separation performance, naphthalene dicarboxylic acid–based Ni-MOF (**NiNDC**, Figure 1) was also prepared and introduced as a filler in 10% weight loading into polycarbonate membranes since 20% of the load did not offer sufficient mechanical properties. The performance of MMMs was also compared with that of other polycarbonate MMMs reported in the literature.

## Materials and Methods

### Materials

All reagents and solvents from commercial sources were used as received without additional purification. Methanol (MeOH, ≥99.9%) and tetrahydrofuran (THF, 99.8%) were purchased from Fischer Chemical, and dimethylformamide (DMF, 99.8%) was purchased from Across Organics. The reagents NiCl_2_·6H_2_O (98%) and 2,6-naphthalene dicarboxylic acid (NDC, 95%) were supplied by Merck and Sigma-Aldrich, respectively. Poly(bisphenol A carbonate-*co*-4,4′-(3,3,5-trimethylcyclohexylidene)diphenyl carbonate) (PC) in pellet form was provided by Aldrich. Its density and glass transition temperature (*T*_g_) were 1.15 g/cm^3^ and 186 °C, respectively; 2,6-di(1*H*-tetrazol-5-yl)naphthalene (NDTz) was prepared according to the procedure reported by us recently.^22^

Gases used in permeation experiments (oxygen, nitrogen, carbon dioxide, hydrogen, methane, and ethylene) were supplied by Praxair with a purity higher than 99.998%.

#### Synthesis of Nickel-Naphthalene Tetrazole MOF (**NiNDTz**)

A solution of NiCl_2_·6H_2_O (26.5 mg, 0.12 mmol) in 0.5 mL of water was added to a 2.0 mL *N*,*N*-dimethylformanide (DMF) solution of 2,6-di(1*H*-tetrazol-5-yl)naphthalene (NDTz) (29.5 mg, 0.12 mmol). The solution was poured into a 4 mL vial and heated at 90 °C for 1 d. Pale pink block-shaped crystals were obtained, which were filtered from the reaction media, washed with DMF, H_2_O, and MeOH, and then dried in air, yielding 40.0 mg of [Ni(NDTz)(H_2_O)_4_]. Calculated: %C, 36.68; %H, 3.59; %N, 28.52; %Ni, 14.9. Found: %C, 37.28; %H, 4.17; %N, 27.28; %Ni, 13.60.

#### Synthesis of Nickel-Dicarboxylic Acid MOF (**NiNDC**)

NiNDC was prepared by a modified method of the previously reported method.^23^ 50 mg (0.231 mmol) of 2,6-naphthalene dicarboxylic acid (NDC) and 55 mg (0.231 mmol) of NiCl_2_.6H_2_O were dissolved in anhydrous DMF (4 mL) and dry methanol (0.6 mL). The mixture was sonicated for 15 min in a pressure tube, heated in an oven at 120 °C for 24 h, and then at 80 °C for 24 h. The green product was washed with DMF, H_2_O, and tetrahydrofuran (THF). Yield: 68.4 mg (77.6%) of [Ni_3_(NDC)_3_(DMF)_2_H_2_O]. Calculated: %C, 38.99; %H, 4.00; %N, 5.05; %, Ni, 31.76. Found: %C, 40.16; %H, 4.20; %N, 2.85; %, Ni, 32.59.

#### Mixed-Matrix Membranes (PC@NiNDTz-10%, PC@NiNDTz-20%, and PC@NiNDC −10%)

The amount of polycarbonate (PC) necessary to prepare the desired hybrid membrane was first dissolved in 5 mL of chloroform. Specifically, 40 or 80 mg (10 or 20% weight) of the corresponding Ni-MOF was dispersed in the above solution containing 360 mg and 320 mg, respectively, by controlled addition of the fillers, in small portions. After the addition of each portion, the solutions were stirred ultrasonically and magnetically for 10 min each. These cycles were repeated until complete filler addition was achieved (6–8 h), and then the dispersion was stirred overnight. Then, the dispersion was poured on a leveled glass plate provided with a confinement ring to obtain a uniform thickness, and it was covered with a funnel to allow slow solvent evaporation at room temperature. Then, the membranes were removed from the glass and dried in a vacuum, increasing the temperature up to 230 °C at intervals of 30 °C to avoid bubble formation. The membranes were maintained at that temperature under a vacuum overnight. After that, the membranes were cooled to room temperature before characterization. A pristine polycarbonate (PC) membrane was also prepared using a solution of 400 mg of PC in 5 mL of chloroform similar to that described for MMMs

#### Characterization Techniques and Permeation Measurements

Fourier transform infrared-attenuated total reflectance (FT-IR-ATR) spectra were obtained using a Bruker Vertex 70v spectrometer. Spectra were recorded at a resolution of 2 cm^–1^ in the spectral range of 400–4000 cm^–1^. The absorbance of the spectra was normalized to the intensity of the peak at about 1160 cm^–1^. Nitrogen adsorption isotherms were recorded on a Micromeritics ASAP 2020 M surface and porosity analyzer at 77 K. Previously, the samples were degassed for 12 h at 120 °C. Specific surface areas were determined by the Brunauer–Emmett–Teller (BET) technique and the pore size average was determined by density functional theory (DFT) methods. Scanning electron microscopy (SEM) micrographs were obtained using a Hitachi SU-8000 microscope operating at 0.8 and 1 kV for PC and MMMs, respectively. The NiMOFs were directly dispersed on a double-sided adhesive, the membranes were fractured under liquid nitrogen, and the cross-section was observed with a magnification that varied from 2 to 90 K. The thermal stability of NiMOFs and MMMs (TGA) was studied by thermogravimetric analysis using a TQ-500 apparatus (TA Instruments). The experiments were carried out under an air atmosphere at a heating rate of 10 °C min^–1^ to a final temperature of 800 °C. The glass transition temperatures, *T*_g_, were determined by differential scanning calorimetry (DSC) using DSC Q-100 equipment (TA Instruments). The samples were encapsulated in standard aluminum DSC pans and were heated at a scanning rate of 20 °C min^–1^ from room temperature to 250 °C in the first cycle to remove their thermal history. Then, the samples were cooled at the same rate, and the glass transition temperatures of MMMs were calculated from the inflection of the heat flow versus temperature curves in the second heat cycle. The skeletal densities of the fillers and dried films were measured in an Accupyc Helium Pycnometer at 25 °C using around 200 mg of sample and at least 3 times for each sample; bulk density was obtained using an analytical Sartorius balance and isooctane as a liquid of known density. Four different pieces of the hybrid membrane were cut, and each of the pieces was weighed 6 times in air and isooctane. The bulk densities of the porous polymer fillers were estimated from the pore volume determined by the N_2_ adsorption isotherms and the skeletal density of the filler according to eq S1 (SI). The density of the hybrid membranes (ρ_MMM_) was calculated theoretically using eq S2 (SI).

Permeation measurements were performed in an experimental constant volume system described previously.^24^ The permeability (*P*), diffusion (*D*), apparent solubility (*S*) coefficient parameters, ideal selectivity (α), and relative errors Δ were calculated from eqs S4–S8 (SI).

## Results and Discussion

### Synthesis and Characterization of Naphthalene-Based NiMOFs (NiNDTz and NiNDC)

**Figure 2:**
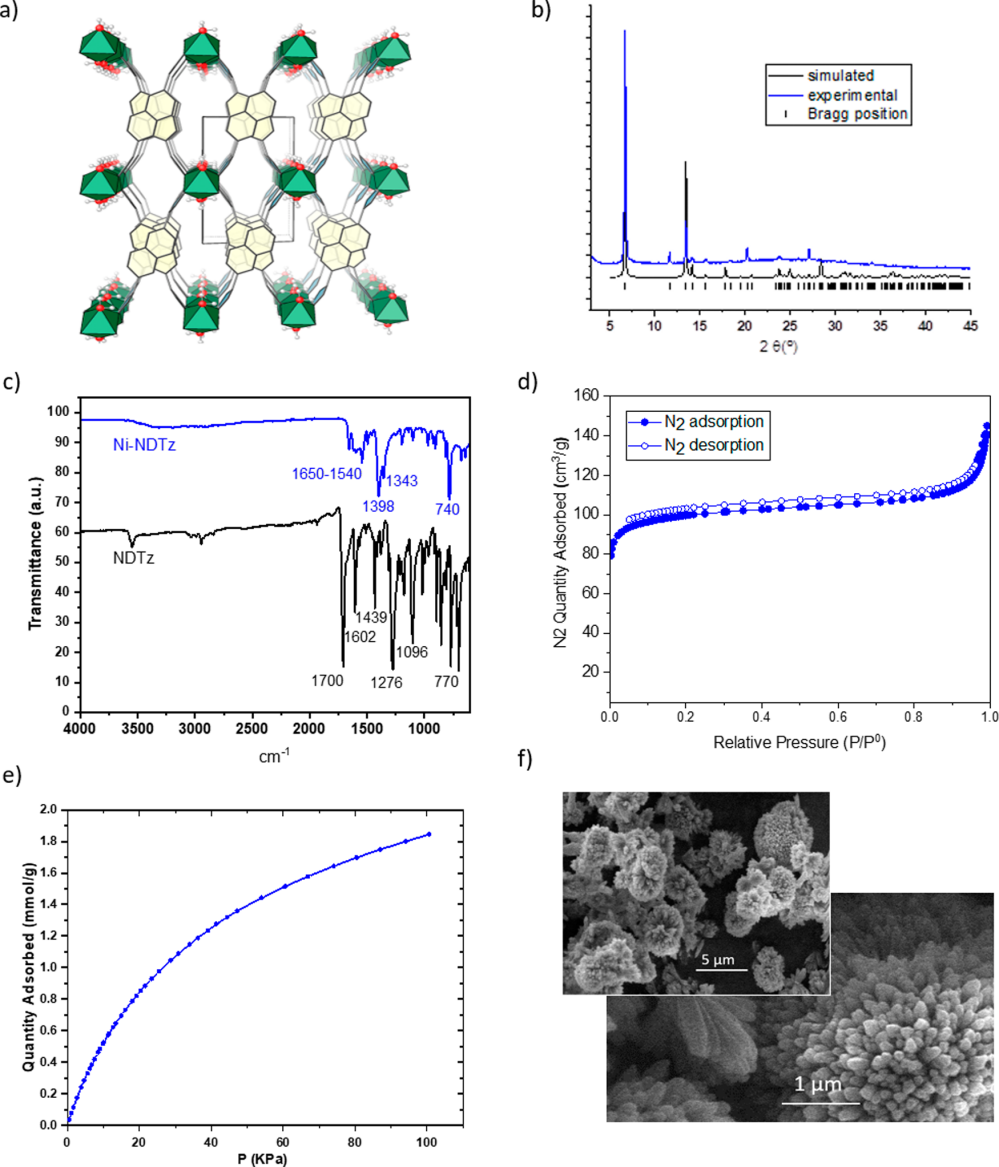
Characterization data of NiNDTz: (a) representation of the building unit formed by coordination of the tetrazolate rings to nickel cations nickel atoms are represented as green polyhedrons, naphthalene as yellow hexagons, tetrazol rings as light-blue pentagons, and oxygen and hydrogen are represented in red and white, respectively; (b) X-ray diffractograms; (c) FT-IR spectra of Ni-NDTz and NDTz linkers; (d) N_2_ adsorption–desorption isotherms; (e) CO_2_ uptake; and (f) SEM images.

The tetrazole-based NiMOF was synthesized by reacting the tetrazole linker, 2,6-di(1*H*-tetrazol-5-yl)naphthalene, with NiCl_2_.6H_2_O, under solvothermal conditions in a mixture of DMF and water, following the analogous protocol reported by our group for cobalt tetrazole MOFs.^22^ The NiNDTz was characterized by elemental analysis, FT-IR, thermogravimetric analysis (TGA), N_2_ adsorption–desorption isotherms, SEM, and powder X-ray diffraction (PXRD). Unfortunately, single crystals of a size suitable for the resolution of the structure by single-crystal X-ray diffraction were not obtained for NiNDTz. Nevertheless, a structural solution was obtained from the analysis of the powder X-ray diffraction (PXRD) pattern. At first sight, the pattern was reminiscent to that of analogous cobalt-NDTz previously reported by our group.^22^ However, the differences in the position of several diffraction peaks were observed, indicating that changes must have occurred in the structure of this MOF. Thus, the PXRD pattern was indexed to the monoclinic system, with unit cell parameters *a* = 8.016 Å, *b* = 13.177 Å, *c* = 7.521 Å, β = 108.84°, and *V* = 752.87 Å^3^. A structural solution was then achieved in the *P* 2/*c* space group, consisting of nickel atoms coordinated to four nitrogen atoms from the tetrazolate linkers and two oxygen atoms from additional water ligands that complete the octahedral geometry. The tetrazolate rings bridge the nickel atoms forming one-dimensional secondary building units that run along the crystallographic *c-*axis and are connected by the organic linkers, resulting in a three-dimensional wine-rack structure (Figure 2a). Figure 2b shows a comparison between the calculated and experimental powder patterns, showing excellent agreement and demonstrating the phase purity of the samples.

The FT-IR (Figure 2c) spectra showed ν(C=N) at 1555 cm^–1^ and bands between 1550 and 1300 cm^–1^ attributed to N=N, C–N, and N–N bonds of the tetrazole rings and the absorption at 740 cm^–1^ of the bond angle deformation of the phenyl rings as previously reported for other compounds containing tetrazole units linked to phenyl rings.^25^ Upon coordination to the Ni(II) centers, there was a red shift in those representative CN bands from the tetrazol ring, observed at 1650–1546 and 1398 cm^–1^.

The thermal stability, analyzed by TGA (Figure S1) in an oxygen atmosphere, revealed high thermal stability up to 300 °C and a degradation pattern in a unique step. Besides, the thermogram shows a weight loss of around 100 °C, which is attributed to the water molecules coordinated to the network. Assuming the formation of NiO (11% according to the TGA residue), the Ni content was determined to be 8.6%. This value and the N content obtained by elemental analysis (see the Materials and Methods Section) indicate that this MOF has a ligand for each Ni metal.

The N_2_ adsorption–desorption isotherm (Figure 2d) showed a typical isotherm with a large N_2_ uptake at low pressure, which also indicates the presence of micropores. The surface area calculated using the Brunauer–Emmett–Teller (BET) method from the nitrogen gas adsorption data was 320 m^2^ g^–1^, which is much higher than that of the CoNDTz recently reported by us.^22^ This result indicates the important role of the metal ion in the development of the porosity of MOFs from tetrazole-based ligands.

The CO_2_ sorption measured at 273 K revealed a high Langmuir CO_2_ specific surface area of 436 m^2^g^–1^ (Figure 2e) and a CO_2_ uptake of 1.85 mmol g^–1^, which was attributed to the tetrazole groups, which are able to interact with CO_2_ through strong dipole–dipole and acid–base interactions, between the protonated and deprotonated forms of the tetrazole ring and carbon dioxide.^26^ Moreover, the high isosteric heat (*Q*_st_= 1.845 kJ mol ^–1^) indicates a favorable interaction between CO_2_ and the network.

The skeletal density and pore volume of the Ni-NDTz MOF, determined by helium pycnometry and N_2_ adsorption isotherms, were 0.89 g cm^–3^ and 0.29 cm^3^ g^–1^, respectively. Therefore, a bulk density of 0.7092 gcm^–3^ can be estimated from expression (1) in SI. As expected, the skeletal density was higher than the bulk density, indicating that the pores of this NiMOF occupied 20% of the total volume of the filler.

The morphology was also studied by SEM (Figure 2f), which revealed elongated oval rough aggregates that made up small, tree-lined bouquets.

**Figure 3:**
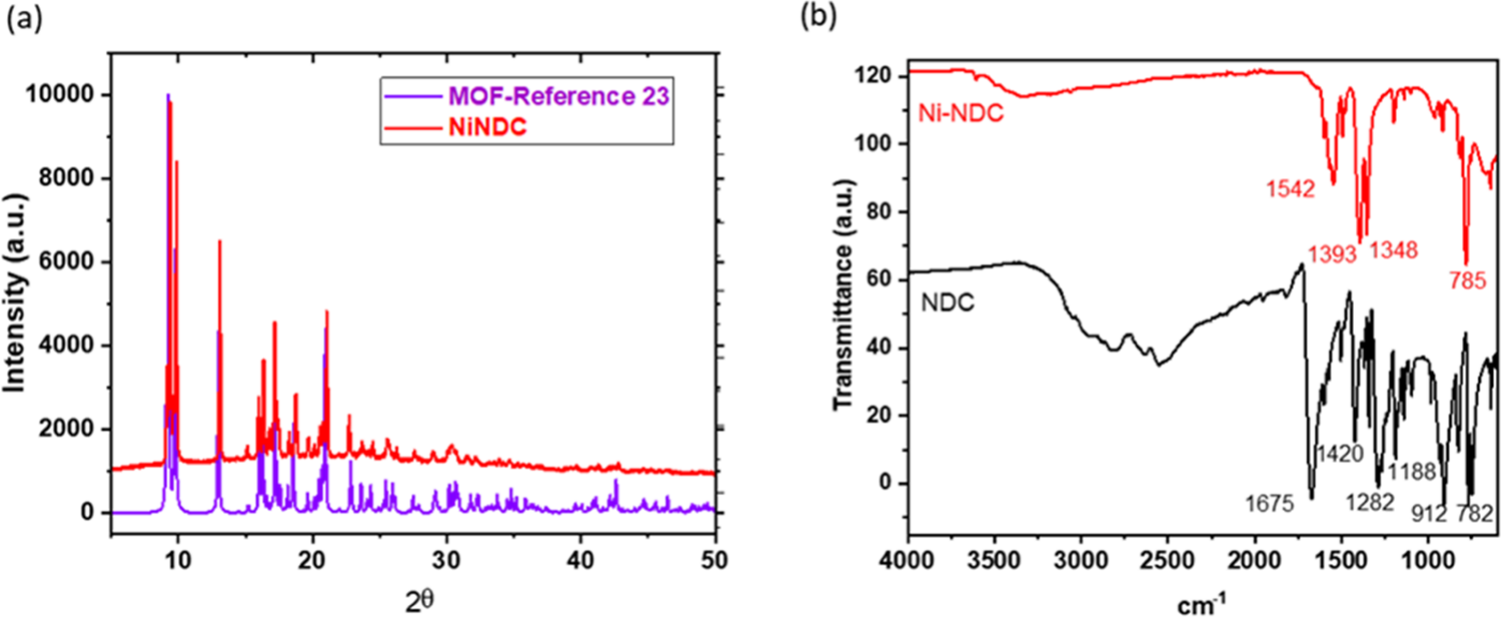
(a) X-ray diffractograms of NiNDC of this work and NiNDC reported. (b) FT-IR spectra of NiNDC and NDC linkers.

The naphthalene-based NiMOF was synthesized from dicarboxylic acid (NiNDC) by adapting the procedure reported by Arrozi et al.^23^ The characterization data of this MOF powder X-ray diffraction (PXRD) (Figure 3a) was similar to that reported for this MOF by Arrozi et al.,^23^ who reported that the carboxylate groups of the ligands are coordinated with N, which completes its coordination sphere with DMF as an amine molecule. The FT-IR spectrum (Figure 3b) shows a carbonyl stretching band at around 1600 cm^–1^. Likewise, a red shift from the typical bands upon the formation of the MOF was observed. Thus, the carbonyl stretching band around 1600 cm^–1^ goes to 1542, and the typical symmetric carboxylate stretching frequency from 1420 goes to 1393 in the NiNDC.

In our case, the coordination sphere was completed with DMF and water molecules [Ni_3_(NDC)_3_(DMF)_2_(H_2_O)_6_], as confirmed by the elemental analysis (see the Materials and Methods Section).

### Preparation of PC-Based Mixed-Matrix Membranes (PC@NiNDTz and PC@NiNDC)

One of the main drawbacks of loading polymeric membranes with MOFs is to achieve good dispersion of the filler in the polymer matrix because of the high tendency of the loads to agglomerate and incompatibility between the two phases. Thus, the incorporation of MOF into polymeric matrices requires a lengthy or tedious treatment. Thus, for example, NiMOF was introduced into an SBS matrix by the preparation of a homogeneous solution of 15% SBS in THF and subsequent addition of this solution to a dispersion of the MOF in THF, with stirring for 2 h, and ultrasonically for 20 min after each addition.^27^

**Figure 4:**
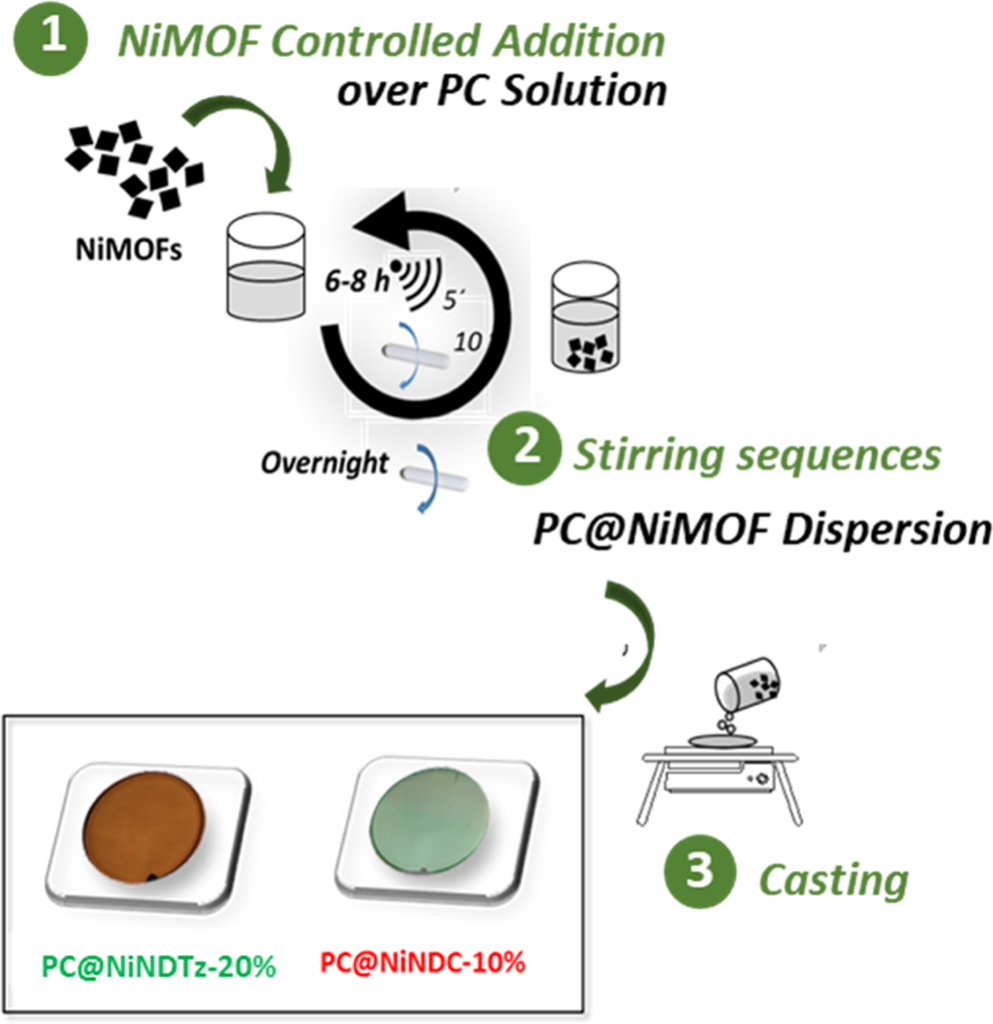
Protocol used in this work to prepare MMMs based on NiMOFs.

In the present work, the preparation of polycarbonate mixed-matrix membranes using naphthalene-based Ni-MOFs was done using an easier protocol (Figure 4), which consisted of the addition of small portions of the corresponding Ni-MOF over a PC chloroform solution under stirring sequences of 5 min ultrasonically and 10 min magnetically after each addition. Once the addition was complete (6–8 h), the mixture was magnetically stirred overnight. After that, the solutions were deposited on leveled glass, and MMMs were obtained by solvent evaporation and drying.

**Figure 5:**
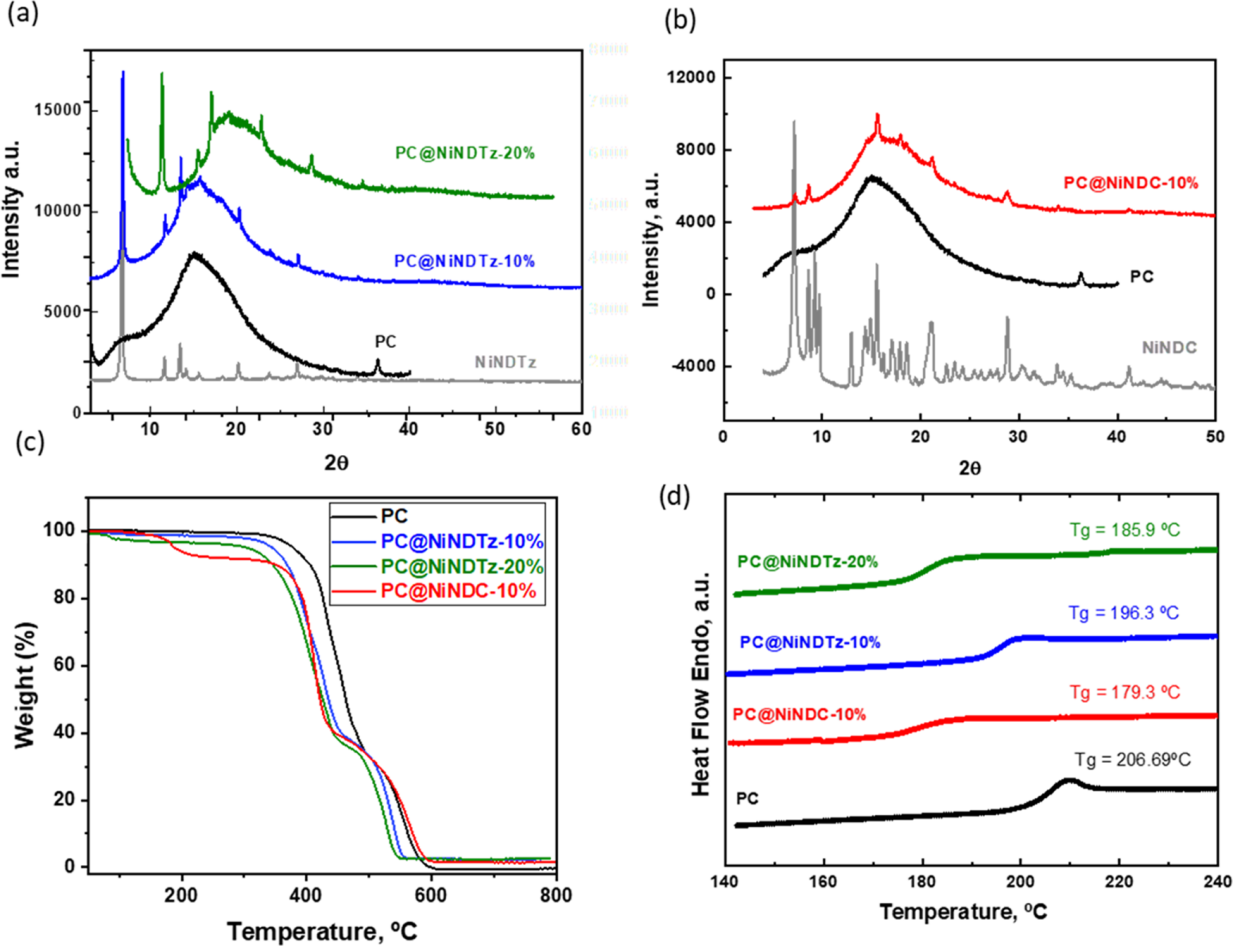
X-ray diffractograms of (a) PC@NiNDTz (10 and 20%) and (b) PC@NiNDC-10% compared to the pristine PC matrix and the corresponding fillers, NiNDTz and NiNDC; (c) TGA analysis and (d) DSC analysis of PC@NiNDTz (10 and 20%) and PC@NiNDC-10% compared to the pristine PC matrix.

The MMMs loaded with 10 and 20% **NiNDTz** were easily removed from the glass and exhibited good mechanical properties, homogeneous appearance, and uniform thicknesses of 106 and 118 μm, respectively. However, the MMM loaded with 20% **NiNDC** exhibited poor mechanical properties, resulting in a brittle membrane not suitable for gas permeation measurements. Attempts were made to prepare MMM on other substrates, and an acceptable membrane was not achieved either. However, a membrane containing 10% **NiNDC** (97 μm) was successfully prepared following the above protocol. These results indicate that the naphthalene dicarboxylic acid linker yields a NiMOF that is much more difficult to disperse in polycarbonate at concentrations above 10% by weight. However, the tetrazole-naphthalene linker has a positive effect on the dispersion of NiMOF in the PC facilitating an increased concentration. Pictures of **PC@NiNDTz-20%** and **PC@NiNDC-10%** films, which exhibit brown and green colors, respectively, are shown in Figure 4. After drying at overnight 230 °C, MMMs were fully characterized. From X-ray diffraction measurements (Figure 5a), it can be observed that the crystalline structure of **NiNDTz** prevails in MMMs prepared with 10 and 20% of this filler, which show the main diffraction peaks at 2θ = 7.77, 11.60, 13.42, 20.32, and 27.04°. Similar behavior was observed for **PC@NiNDC-10%** (Figure 5b), which shows the characteristic peaks of the filler at 2θ = 7.25, 8.61, 15.58, 17.98, 21.24, and 28.72°. Thermogravimetric analyses showed that all NiMOFs decreased the thermal stability of PC. However, the values of Td did not vary significantly as the concentration of the NiNDTz filler in the matrix increased. Comparing the two NiMOFs with the same concentration (10%), it can be observed (Figure 5c and Table S1) that whereas the tetrazol-MOF provokes a decrease of 7% in the decomposition temperature of the pristine matrix, the dicarboxylic acid ligand makes the PC more unstable at about 5%. PC degradation occurs in one step, while MMMs undergo two degradation steps. This could be attributed to the presence of NiMOF. Thus, the first step would be due to the degradation of the linear PC fractions and the second would be due to the cross-linked dispersed fractions containing NiMOF and PC.

The glass transition temperature (*T*_g_) determined by DSC (Figure 5d and Table S1) decreased with the addition of NiNDTz to the polycarbonate. The glass transition temperature is lower in confined geometries than in bulk because the α relaxation, associated with the glass transition is faster, favoring chain movement.^28^ Thus, as the amount of filler increases, there is a greater probability that the polymer chains can enter the pores, which would partially confine the PC chains and, therefore, reduce the *T*_g_ of the mixed-matrix membranes. It seems that PC also has a great tendency to enter the pores of NiNDC since the Tg of the matrix also decreases when loaded with 10% MOF. In fact, the density values calculated using eq S2 in SI were significantly lower than the experimental values (Table S1). Assuming that the pores of NiNDTz are totally filled by the polymer chains, the fraction of polycarbonate that does not occupy these pores (ω_PC_) can nearly be determined from expression 8 (SI). Thus, the fractions of PC in the NiNDTz pores were 0.16 and 0.33 for **PC@NiNDTz-10%** and **PC@NiNDTz-20%**, respectively, which are in accordance and could explain the decrease in the *T*_g_ values of the mixed-matrix membranes with respect to PC since the polymer chain movements would be less restricted.

**Figure 6:**
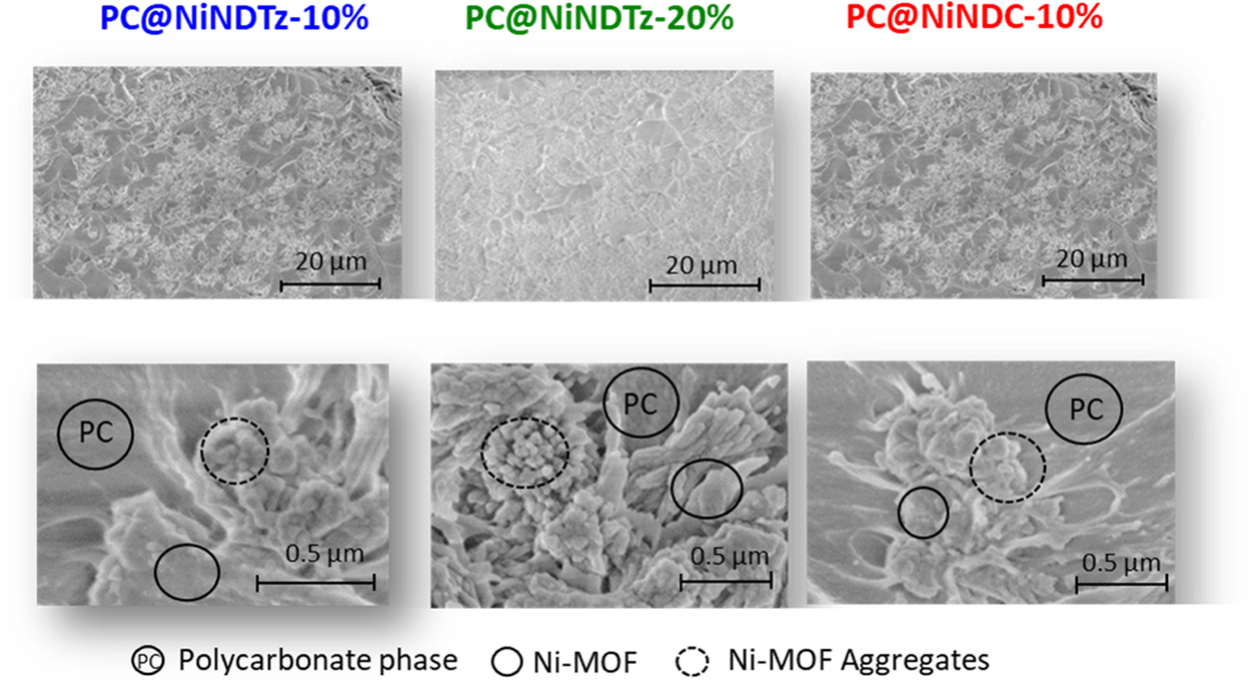
SEM images of the cross-sections of PC@NiMOFs at 5K (20 μm scale) and 60K magnifications (0.5 μm scale).

The SEM images of the MMMs at 5K magnification (20 μm scale) (Figure 6) showed good distribution of NiMOF in the polymeric phase although some degree of filler aggregation is noticed in all cases. However, it appears that the size of the NiNDTz dispersed in the polycarbonate matrix decreased in the 20% film. This reduction in particle size could be attributed to the effect of the preparation process of the membrane itself, which required a longer overall sonication time in order to decrease the aggregation of the neat filler, which would lead to a reduction in its size. It is also important to note the good compatibility between NiMOF fillers and polymer matrix due to the apparent absence of defects or voids in the interface of all hybrid membranes, as can be seen in the SEM images in Figure 6 at 60K magnifications (0.5 μm scale)

### Gas Separation Properties

Considering that gas transport through a dense membrane can be explained by a diffusion-solution mechanism that takes place in three steps (the sorption of the permeant in the membrane, its diffusion through it, and the desorption of the gas at the other side of the film), the permeability can be expressed as a product of a kinetic parameter (diffusion coefficient) and a thermodynamic one (solubility coefficient). In this case, the evolution of the pressure of gas, *p*(*t*), as a function of time can be deduced by the integration of Fick′s second law using appropriate boundary conditions according to eq S3 in SI.

The permeability coefficients (*P*) were measured at 30 °C and 1 bar for different gases in the following order: O_2_, N_2_, CO_2_, H_2_, CH_4_, and C_2_H_4_. In all hybrid membranes prepared in this work, the variation of the gas pressure in the downstream chamber fits very well with eq S3 (SI), as can be seen in Figure S2 (SI). For example, the values of permeability and diffusion coefficients of oxygen and nitrogen in the membrane PC@NiNDTz-20%, obtained by fitting this equation to the experimental results, were 8.3 barrer and 7.4 × 10^–8^ cm^2^ s^–1^ for oxygen and 1.7 barrer and 1.9 × 10^–8^ cm^2^ s^–1^ for nitrogen. These values are the same as those obtained from the time dependence of the gas pressure in the steady state conditions (eqs S4 and S5 in SI (Table S2)).

**Figure 7:**
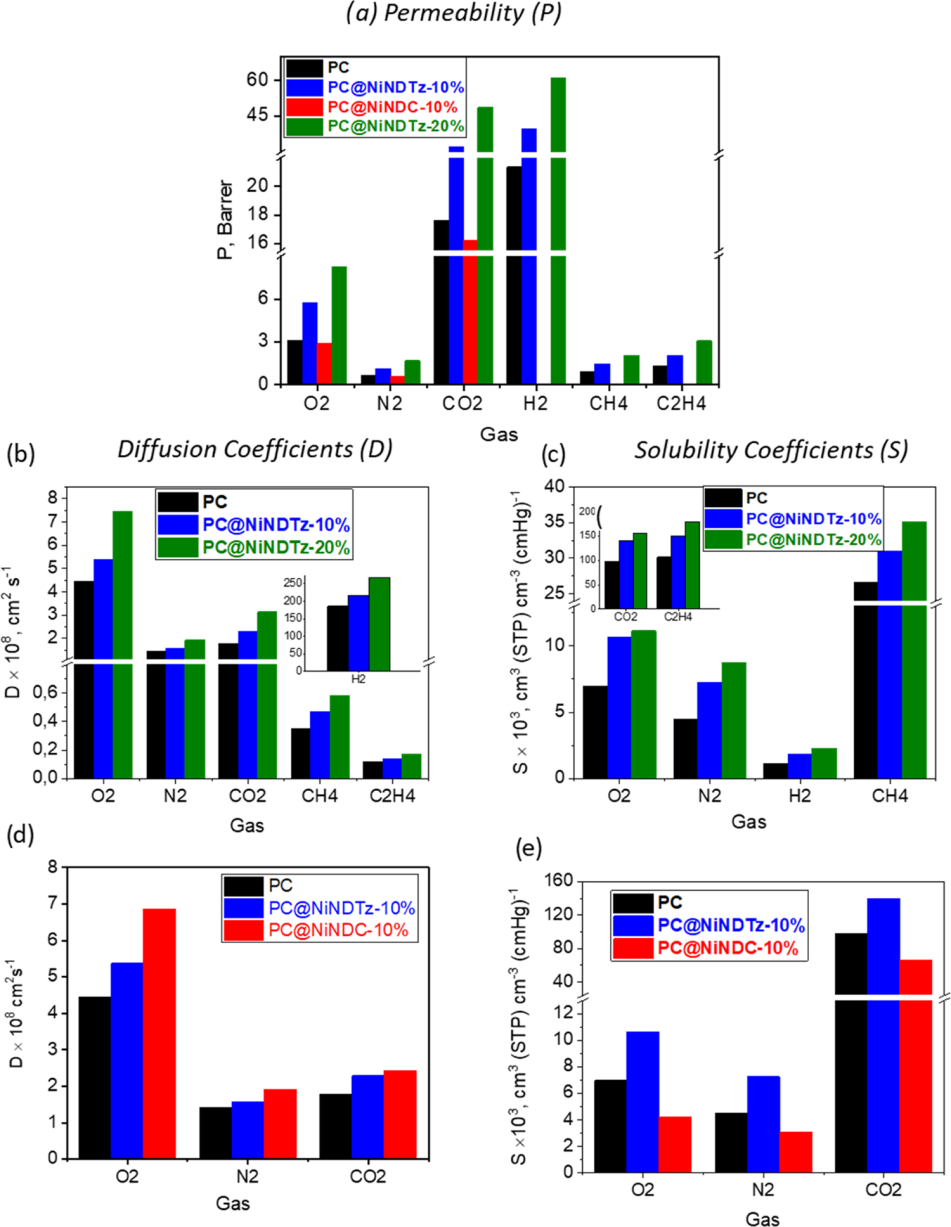
Graphic representations of (a) permeability coefficients, (b, d) diffusion, and (c, e) apparent solubility coefficients, for the pure polycarbonate membrane (PC) and the corresponding MMMs at 30 °C and 1 bar pressure.

To establish the effect of the tetrazole linker on gas separation properties, the polycarbonate-based membrane **PC@NiNDTz-10%** was initially compared with that prepared using a dicarboxylate linker, **PC@NiNDC-10%** (Figure 7a and Table S2). The first important difference was observed in the mechanical properties during the permeation tests. The **PC@NiNDC-10%** membrane was broken after the permeation of CO_2_ while **PC@NiNDTz-10%** maintained its mechanical properties after the permeation of all gases. This showed a positive effect of the tetrazole groups, which should have a more favorable interaction with the matrix. Moreover, the addition of the NiNDC filler to polycarbonate did not have any significant influence on the permeability coefficients of the measured gases, which indicates that 10% of this filler is not enough to induce changes in the gas transport properties of PC. However, **PC@NiNDTz-10%** increased the P values of neat **PC** by up to 1.80 times (85%) on the studied gas and **PC@NiNDTz-20**% increased the values by up to 2.8 times (185%) (see Table S2).

An increase in permeability with loading has already been observed for other porous fillers in other matrices in gas separation membranes.^29^ The balance between *D* and *S* will determine the permeability of a gas in a polymer matrix or a mixed matrix. As stated above, permeability (*P*) is a contribution of kinetic and thermodynamic parameters, *D* (diffusion coefficient) and *S* (solubility coefficient), respectively. The first is related to the available free volume, the polymer chain mobility, and the kinetic diameter of the gas, while the solubility coefficient is influenced by gas condensability, gas–polymer interactions, and nature of the membrane (glassy, elastomers, or rubber polymers).^30^ Thus, *D* and *S* parameters were also determined (Figure 7b–e and Table S2) to understand the increases in permeability in the MMMs. Compared with pure PC, the diffusion coefficients (Figure 7b and Table S2) increased around 1.2 times (28%) in the case of **PC@NiNDTz-10**% and around 1.7 times (72%) in the case of **PC@NiNDTz-20**%, while the solubility coefficients increased a little more, around 1.5 times (51%) in the case of **PC@NiNDTz-10**% and 1.9 times (95%) in the case of **PC@NiNDTz-20**%. These results indicate that the increase in permeability is a contribution of both kinetic and thermodynamic parameters, with the latter having a slightly higher contribution. The slight increase in *D* for **PC@NiNDTz-10**% (1.1 times) compared to pure PC indicated that this amount was not enough to alter the PC chain movement. However, when 20% w/w NiNDTz was added to the PC, the diffusion coefficient increased 1.5-fold, suggesting that this amount of filler does cause an increase in the distance between the polymer chains since the formation of holes between the filler and the bare polycarbonate was not observed in the SEM images (see Figure 6). As expected, the diffusion coefficients followed the expected trends: *D*(H_2_) ≫ *D*(O_2_) > *D*(CO_2_) > *D*(N_2_) > *D*(CH_4_) > *D*(C_2_H_4_) in agreement with the kinetic diameters of the gases.

The increases in the solubility coefficients (Figure 7c and Table S2) were greater than those of diffusion and were appreciable in both membranes being higher in the membrane with 20% filler, which indicates that not only the nature of the filler but also its concentration improves the interactions with the gas. Furthermore, the pores of this NiMOF, with an average diameter of 26.2 Å, would probably allow the adsorption of small molecules of gases inside. That is to say, the interior of the NiNDTz crystalline units can provide Langmuir sites where adsorption processes may take place, as has been described for MMM prepared with ZIF-8 as a filler and polyether ether sulfone as a polymer matrix.^31^

As expected, *S* was greater in the most condensable gases such that *S*(C_2_H_4_) > *S*(CO_2_) > *S*(CH_4_) > *S*(O_2_) > *S*(N_2_) > *S*(H_2_). Comparing the *D* and *S* coefficients of **PC@NiNDTz-10**% with **PC@NiNDC-10**%, for the gases that could be measured in the two membranes (Figure 7d,e and Table S2), it can be observed that *D* slightly increases with respect to pure PC and the values are similar for both types of fillers. However, *S* only increased in the case of the tetrazole-based linker, indicating that the interaction between the gases and MMM was clearly favored by the presence of the tetrazole groups.

### Performance Evaluation of PC@NiNDTz

The real performance of the gas separation membrane is established by analyzing its permeability versus selectivity. A good compromise is to improve the gas flux through the membranes (increase the permeability coefficients) without loss of selectivity. This is really a difficult target because when one parameter increases, the other decreases and vice versa. The mixed membranes not only maintain the selectivity of pure PC (Table 1) but also increase it for the following gas pairs: CO_2_/CH_4_, CO_2_/C_2_H_4_, H_2_/CH_4_, and H_2_/C_2_H_4_. These results indicate that NiNDTz is a very good filler to improve the processes that involve CO_2_ and H_2_ purification. In particular, the separation of CO_2_ from methane and ethylene in **PC@NiNDTz-20%** showed improvements of 24 and 15%, respectively, compared with pure PC. Regarding hydrogen recovery, the improvements in the separation of the same gases were 28 and 19%, respectively. These results indicate that **PC@NiNDTzs** are very productive membranes for CO_2_ and H_2_ recovery, overcoming the PC membrane.

**Table 1:** Selectivities of PC and PC@NiNDTz for Different Pairs of Gases

membrane	O_2_/N_2_	CO_2_/N_2_	CO_2_/CH_4_	CO_2_/C_2_H_4_	H_2_/CO_2_	H_2_/CH_4_	H_2_/C_2_H_4_	C_2_H_4_/CH_4_
PC	4.8	27.5	19.1	13.7	1.2	23.2	16.7	1.4
PC@NiNDTz-10%	5.0	28.1	21.9	15.4	1.2	27.2	20.5	1.3
PC@NiNDTz-20%	4.9	29.0	23.7	15.8	1.3	29.7	19.9	1.5

**Figure 8:**
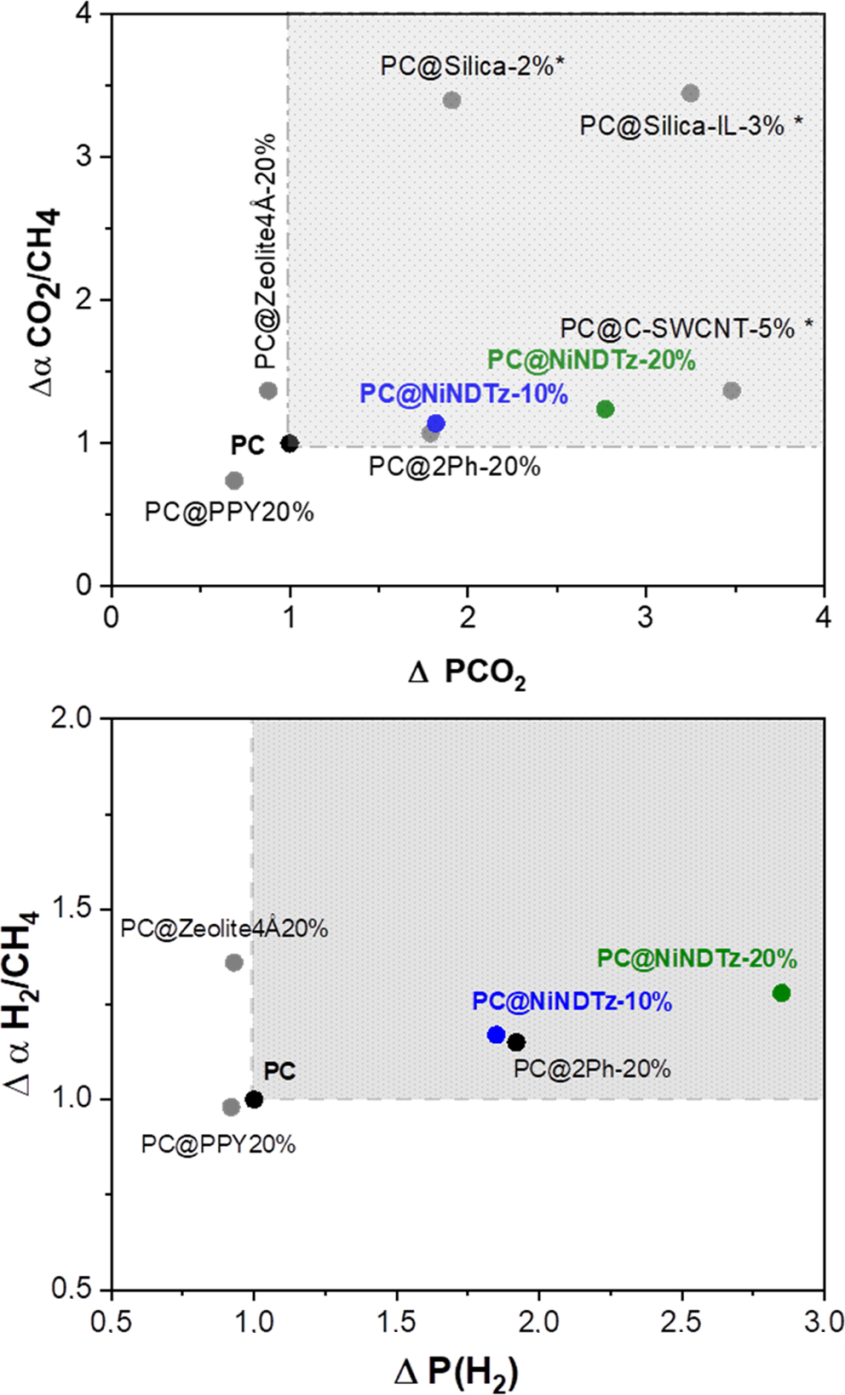
Graphic representation of the variations in selectivity and permeability of PC@filler hybrid membranes (reported and those of this work) with respect to pure PC for CO_2_/CH_4_ (a) and H_2_/CH_4_ (b) gas pair. Gray box: MMMs that improve the PC membrane performance. * Measured at 2 bar.

To evaluate the performance of the MMMs, the above results were compared with those reported for PC-based MMMs containing other porous fillers, measured at conditions similar to those reported by us. The membranes selected for comparison were the best of the series reported. Thus, the gas separation performances of **PC@NiNDTz-10%** and **PC@NiNDTz-20%** were graphically represented with the mixed-matrix membranes selected from the literature (Figure 8), which were designed with the name of the matrix, PC, followed by the name, and the amount of the filler (PC@Filler%). Those inside the gray zone improved the performance of polycarbonate (PC). For CO_2_/CH_4_ separation (Figure 8a), the hybrid membranes containing silica (PC@Silica-2%) and silica filler modified with ionic liquid PC@Silica-IL-3% are the membranes with the best performance, although the data reported are measured at 2 bar.^32^ The incorporation of 5% of a functionalized carbon nanotube with the carboxylic group yields an MMM, PC@C-SWCNT5%, with good performance for this separation since the *P*(CO_2_) coefficient increased 3.5-fold with respect to pure PC and a selectivity around 1.2-fold although the experiments were also carried out at 2 bar.^33^**PC@NiNDTz-10%** showed a similar performance to that of **PC@2Ph-20%**, a hybrid membrane recently reported by us^24^ with 20% of a porous organic polymer containing exclusively carbon and hydrogen atoms and with a specific surface area of 1481 m^2^ g^–1^. This shows that fillers with very different structures and compositions can provide membranes with the same productivity and confirms the important role of tetrazole groups in the gas separation properties of MMMs prepared in this work. In fact, the selected MMM containing polypyrrole, an N-based filler (PC@PPY-20%), did not improve the PC performance,^34^ which confirms that the structure of tetrazole is decisive in improving the gas separation properties. The number of these groups is also very important since **PC@NiNDTz-20%** was better positioned in the graphic than MMM with 10% of this filler. The selected membrane containing zeolite as a filler did not improve the gas transport properties of the PC.^35^

Regarding H_2_/CH_4_ separation, there are a lot of matrices loaded with porous fillers to achieve this separation. However, using PC as a matrix, few examples have been found in the literature (Figure 8b). The addition of PPY (polypyrrole chemically synthesized) as a filler maintained the selectivity of PC, whereas zeolite 4 Å increased it 1.4 times.^34,35^ However, the P coefficients for H_2_ did not surpass that of PC. As in the above separation, **PC@2Ph-20%**(24) and **PC@NiNDTz-10%** exhibited similar performance. Compared with the few published data addressing this separation with PC-based MMMs, **PC@NiNDTz-20%** showed the best performance for H_2_/CH_4_ separation, clearly improving the gas separation properties of pure PC.

Although NiMOFs have not been used as fillers in polycarbonate membranes, they have been used as fillers in other matrices for CO_2_/CH_4_ separation (Table 2).^36−38^ Comparing the two NiMOFs that produced the greatest increases in permeability, [Ni_3_(OH)_2_(1,4-BDC)_2_- (H_2_O)_4_]·2H_2_O (6%) and NiMOF in this work, NiNDTz (20%), it should be noted that the latter produced a greater increase in both permeability and selectivity parameters. The increase in the permeability generated by this filler supports the affinity of the tetrazole linker for CO_2_.

**Figure 9:**
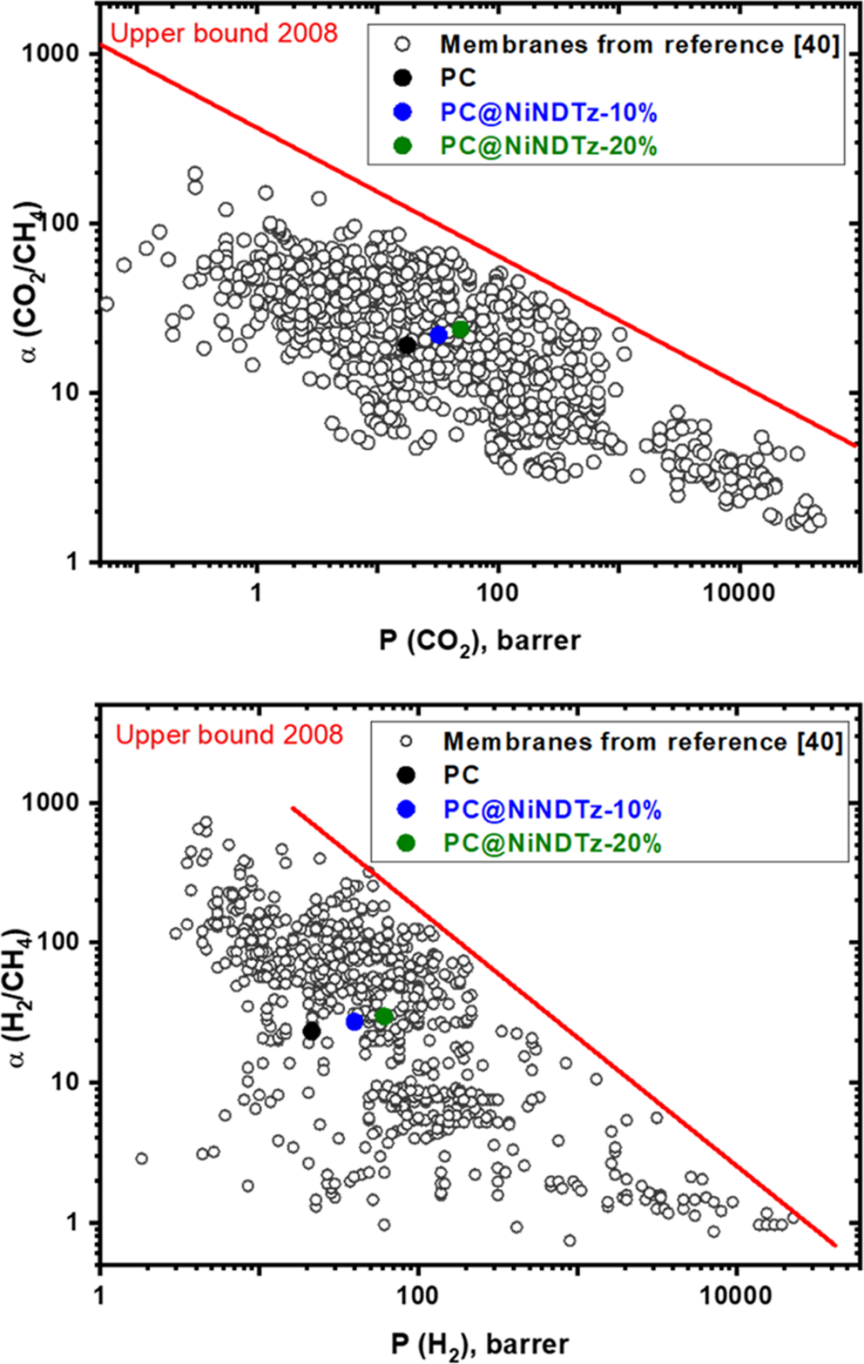
Performances of PC@NiNDTz-10%, PC@NiNDTz-20%, and polycarbonate (PC) membranes for CO_2_/CH_4_ (up) and H_2_/CH_4_ (down) separations depicted in Robeson-type diagrams.

Besides, in order to evaluate the general performance of the MMMs, CO_2_/CH_4_ and H_2_/CH_4_ selectivities were plotted versus CO_2_ and H_2_ permeabilities, respectively, in Robeson-type diagrams (Figure 9).

The mixed-matrix membranes, PC@NiNDTz-10% and PC@NiNDTz-20%, are closer to the upper limit than the polycarbonate membrane, which indicates greater productivity in the separation of these pairs of gases for these hybrid membranes. However, the productivity is lower than many of the reported membranes,^39,40^ although it must be considered that most of them are based on polyimides (PIs) and polymers of intrinsic porosity (PIMs).

**Table 2:** Increases in *P*(CO_2_) and α(CO_2_/CH_4_) of NiMOF-Based MMMs Reported in the Literature and in This Work^a^,^b^,^c^,^d^,^e^

NiMOF	load (%)	matrix	increase in *P*(CO_2_) (%)	increase in α(CO_2_/CH_4_) (%)	refs
[Ni_3_(OH)_2_(1,4-BDC)_2_-(H_2_O)_4_]·2H_2_O	6	Pebax1657	115	15	(36)
Ni-NH_2_-BDC	5	Pebax1657	66	192	(37)
KAUST-7	33	6FDA-Durene	36	50	(38)
NiNDTz	10	PC	85	15	this work
NiNDTz	20	PC	177	24	this work

a BDC: 1,4-benzenedicarboxylate.

b Pebax1657: polyether-block amide.

c KAUST-7: NbOFFIVE-1-Ni.

d 6FDA: 4,4-(hexafluoroisopropylidene) diphthalic anhydride.

e Durene: 2,3,5,6- tetramethyl-1,4-phenylenediamine.

## Conclusions

We have found that a tetrazole-naphthalene-Ni-based-MOF (NiNDTz) has good compatibility and better dispersion properties in a polycarbonate matrix than the corresponding naphthalene dicarboxylic acid Ni-based-MOF (NiNDC), resulting in MMMs with suitable mechanical properties and homogeneous filler distribution. In fact, it was only possible to prepare a membrane with 10% NiNDC, which only resisted the permeation of three gases: O_2_, N_2,_ and CO_2_. Compared with neat PC, the **PC@NiNDTzs** increased the permeability coefficients of all gases by up to 2.8 times (185%), depending on the filler content and gas tested. The increase in permeability was attributed to the kinetic (diffusion) and thermodynamic (solubility) parameters, although the contribution of the latter was much greater and was clearly attributed to the presence of the tetrazole groups. The existence of cavities or Langmuir sites in MMMs reported in this work where gases can be adsorbed needs to be verified; therefore, sorption experiments are currently being conducted to probe it. Besides, the mixed membranes increase the selectivity in separations where CO_2_ or hydrogen are involved. In particular, the performance of **PC@NiNDTz-20%** was especially relevant since improvements of 24 and 15% in the separation of CO_2_ from methane and ethylene, respectively, were achieved, as well as for the separation of hydrogen from the same gases where the selectivities were increased by 28 and 19%, respectively. With this membrane, an increase in the permeability of CO_2_ and H_2_ close to 3 times and improvements in selectivity between 24 and 28% were obtained, which is quite an achievement in the field of gas separation since improvements have been achieved in both parameters simultaneously. In comparison with other PC-based MMMs reported in the literature, we can conclude that silica and silica-ionic liquid fillers exhibit better performance for CO_2_/CH_4_ separation than the NiNDTz filler in this work. However, for H_2_/CH_4_ separation, which is of great interest in the petrochemical industry, **PC@NiNDTz-20%** is the best membrane reported to date to carry out this separation using polycarbonate as a matrix. In comparison with other gas separation membranes, the MMMs based on NiNDTz are distant from the 2008 Robeson upper bound. In comparison with other NiMOF-based MMMs, NiNDTz (20%) in a polycarbonate matrix generated the greatest increase in CO_2_ permeability.

## Supporting Information Available

Thermogravimetric analysis of NiNDTz (Figure S1); experimental (scatters) and calculated (continuous lines) pressure values of oxygen and nitrogen in the downstream chamber as a function of time in the PC@NiNDTz-20% membrane at 30 °C and 1 bar of pressure (Figure S2); thermal properties and density of polycarbonate (PC) and PC@NiMOF films of this work (Table S1); values of the permeability, *P*, diffusion, *D*, and apparent solubility, *S*, coefficients for oxygen, nitrogen, carbon dioxide, hydrogen, methane, and ethylene at 30 °C and 1 atm of polycarbonate (PC) and PC@NiMOF films of this work (Table S2) (PDF)
