# Supplementary material for: Naphthalene Tetrazole-Based Nickel Metal–Organic Framework as a Filler of Polycarbonate Membranes to Improve CO2 and H2 Separation
Source: ACS Appl Polym Mater. 2024 Apr 1;6(7):4244–55. doi: 10.1021/acsapm.4c00277 (PMC11019729; doi:10.1021/acsapm.4c00277)
Supplement: Supplementary file 1 — ap4c00277_si_001.pdf [file ap4c00277_si_001.pdf]

Supporting Information

Naphthalene tetrazole-based Nickel Metal Organic Framework as filler of polycarbonate membranes to improve CO_2 and H_2 separations

Antonio Valverde-Gonzalez,[a] *Nastasiya Yuriychuk,*[b] *M Carmen Borrallo-Aniceto,*[a] *Felipe Gandara,*[a] *Marta Iglesias,*[a] *Mar López-González*[b,*] *and Eva M. Maya,*[a,*]

[a] Departamento de Fronteras en Química de Materiales, Instituto de Ciencia de Materiales de Madrid (ICMM), CSIC, Sor Juana Inés de la Cruz, 3, Cantoblanco. Madrid 28049, Spain. e-mail: eva.maya@csic.es,

[b] Departamento de Química-Física de Polímeros, Instituto de Ciencia y Tecnología de Polímeros (ICTP-CSIC), Consejo Superior de Investigaciones Científicas, C/ Juan de la Cierva 3, Madrid 28006, Spain. e-mail: mar@ictp.csic.es

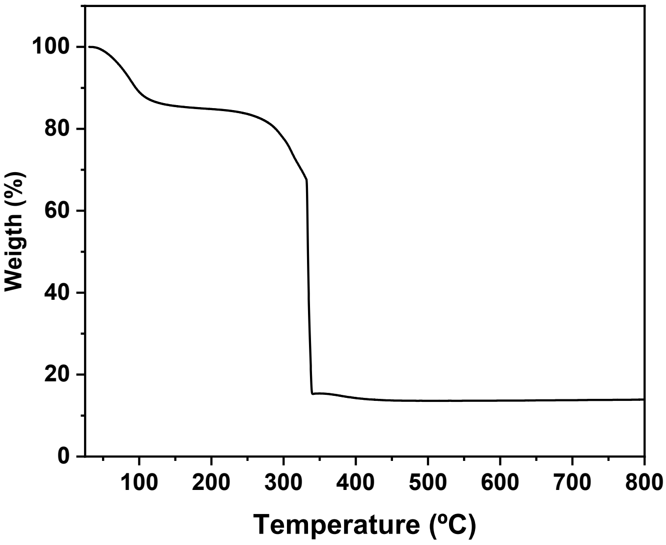

Figure S1. Thermogravimetrical analysis of NiNDTz

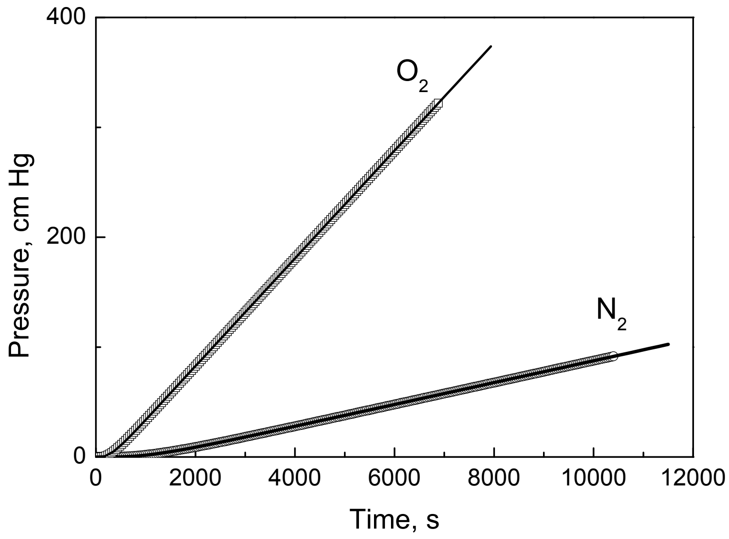

Figure S2. Experimental (scatters) and calculated (continuous lines) values of the pressure of oxygen and nitrogen in the downstream chamber as a function of time in PC@NiNDTz-20% membrane at 30 °C and 1 bar of pressure

Table S1. Thermal properties and density of polycarbonate (PC) and PC@NiMOFs films of this work.

Membrane	Td (˚C)	Tg (°C)	Skeletal Density (g/cm^3)	Bulk Density (g/cm^3)	
				Experimental	Calculated[a]
PC	433; 460	206	--	1.150[b]	
PC@NiNDTz-10%	400; 428	196	0.9355	1.1465	1.0827
PC@NiNDTz-20%	404; 433	186	1.0495	1.1598	1.0228
PC@NiNDC-10%	412	179	1.1033	1.2391	n.r.[c]

[a] Calculated from equation (1) in SI [b] Value supplied by Aldrich [c] not reported because the pore volume of NiNDC is not available.

Table S2. Values of the permeability, *P*, diffusion, *D*, and apparent solubility, *S*, coefficients for oxygen, nitrogen, carbon dioxide, hydrogen, methane and ethylene at 30 °C and 1 atm of pressure for the MMMs made from PC

Membrane	Gas	P, [a]	$D \times 10^8$ [b]	$S \times 10^3$ [c]
	O_2	3.1	4.5	6.9
	N_2	0.6	1.4	4.3
**PC**	CO_2	17.6	1.8	97.9
(97 ± 1)	H_2	21.4	186.2	1.15
	CH_4	0.9	0.4	26.6
	C_2H_4	1.3	0.1	107.0
	O_2	5.7	5.4	10.6
	N_2	1.1	1.6	7.2
**PC@NiNDTz-10%**	CO_2	32.1	2.3	147.8
(106 ± 1)	H_2	39.7	216.8	1.8
	CH_4	1.1	0.5	31.0
	C_2H_4	2.1	0.1	139.5
	O_2	2.9	6.8	4.2
**PC@NiNDC-10%**	N_2	0.6	1.9	3.1
(97 ± 1)	CO_2	16.2	2.4	66.5
	O_2	8.3	7.4	11.1
**PC@NiNDTz-20%**	N_2	1.7	1.9	8.7
(138 ± 4)	CO_2	48.8	3.1	155.8
	H_2	61.1	267.6	2.3
	CH_4	2.1	0.6	35.1
	C_2H_4	3.1	0.2	180.5

a) 1 Barrer= 10^{-10} cm^3 (STP) cm cm^{-2} s^{-1} $(cmHg)^{-1}$
b) D in cm^2 s^{-1}
c) S in cm^3 (STP) cm^{-3} $(cmHg)^{-1}$

Equations

Bulk density is a characteristic of the material that includes the volume of the compact solid material, the volume of both closed and open pores and the interparticle voids. For a porous polymer filler, bulk density in g.cm^{-3}, were estimated using the following equation:

$$\frac{1}{\rho_{bulk\ density}} = V_{pore} + \frac{1}{\rho_{skeletal}} \qquad (1)$$

Where V_{pore} is the pore volume determined by N_2 adsorption isotherms.

Bulk density of the hybrid membranes were calculated theoretically by the following expression

$$\rho_{MMM} = \frac{\rho_{MOF}\,\rho_{PC}}{\omega_{MOF}\,\rho_{PC} + (1-\omega_{MOF})\rho_{MOF}} \qquad (2)$$

where ω_{MOF} is the mass fraction of NiMOF filler in the MMMs whereas ρ_{MOF} and ρ_{PC} are the bulk densities of MOF filler and PC, respectively.

Gas transport through a dense membrane can be explained by a diffusion-solution mechanism according to which the evolution of the pressure of permeant (in cm Hg) with time is given by [Crank, J. *The Mathematics of Diffusion*, Oxford University Press, 1975]

$$p(t) = 0.2786\frac{p_0 ALST}{V}\left(\frac{Dt}{L^2} - \frac{1}{6} - \frac{2}{\pi^2}\sum_{n=1}^{\infty}\frac{(-1)^n}{n^2}\exp\left(-\frac{Dn^2\pi^2 t}{L^2}\right)\right) \qquad (3)$$

where p_0 is the pressure in cm Hg of the gas at the upstream chamber, the solubility S and diffusion *D* coefficients are given, respectively, in [cm^3(STP) /cm^3 cm Hg] and $cm^2 s^{-1}$, A and L are, respectively, the area in cm^2 and the thickness in cm of the film, and V is the volume in cm^3 of the downstream chamber.

In the steady state, equation 3 can be simplified and a linear function of time is found for the gas pressure in the downstream chamber. So, permeability coefficients, *P*, can be obtained from:

$$P = \frac{273}{76}\frac{Vl}{p_0 TA}\lim_{t\to\infty}\frac{dp}{dt} \qquad (4)$$

where V and *l* are the volume of the downstream chamber in cm^3 and thickness in cm of the membrane, respectively, *T* is the absolute temperature in K, *A* is the diffusion area in cm^2, p_0 and p are the upstream and downstream gas pressures in cmHg, respectively. *P* is given in Barrer [1 Barrer=10^{-10} cm^3 (STP) cm cm^{-2} s^{-1} $(cmHg)^{-1}$]

The diffusion coefficient, *D*, in cm^2 s^{-1} can be obtained from the time lag (θ) using the following equation:

$$D = \frac{l^2}{6\theta} \qquad (5)$$

The apparent solubility coefficient (in cm^3 (STP) cm^{-3} $(cm\ Hg)^{-1}$) can be calculated from P and D with the expression:

$$S = P/D \qquad (6)$$

The ideal selectivity that a membrane has to separate two gases is defined as the ratio

$$\alpha\left({}^{A}/{}_{B}\right) = \frac{P(A)}{P(B)} \qquad (7)$$

where *P(A)* and *P(B)* are the permeability coefficients of gases A and B, respectively. In all cases *P(A) > P(B).*

Finally, the relative errors Δ involved in the determination of D by the time lag method were calculated by means of the following expression

$$\Delta = \left(\frac{|L\,\epsilon(L)|}{3\,\theta} + \frac{|\epsilon(\theta)|\,L^2}{6\,\theta^2}\right)/D \qquad (8)$$

In all experiments, this error Δ lied in the range of 2 to 3% whereas for the permeability coefficients the standard deviation was lower than 2%.

If we assume that the pore of the fillers is totally filled by the polymer chains, the fraction of polycarbonate not occupying these pores, ω_{PC}, can nearly be determined from the following equation:

$$\omega_{PC} = \frac{\rho_{PC} \;\; \rho_{MOF} - \omega_{MOF}\rho_{MMM}}{\rho_{MMM} \qquad\quad \rho_{MOF}} \qquad (9)$$

Where ρ_{PC}, ρ_{MOF} and ρ_{MMM} are the densities of the pure polycarbonate, the filler and the mixed matrix membranes, respectively, and ω_{MOF} is the molar fraction of filler MOF in the MMM
